# The Role of the Indian Political Regime in Higher Education Reforms for Innovation Drive: Key Comparisons With China

**DOI:** 10.1177/00219096221097666

**Published:** 2022-06-07

**Authors:** Romi Jain, Eric Ping Hung Li, Joseph Tse-Hei Lee

**Affiliations:** Faculty of Management, The University of British Columbia- Okanagan Campus, Canada; Department of History, Pace University, USA

**Keywords:** China, higher education, India, knowledge economy, innovation

## Abstract

As primary drivers of global growth, China and India as Asian giants are on the path to reforming their higher education systems to drive innovation. This paper based on both primary and secondary data sources investigates how India’s democratic political leadership has facilitated higher education reform for fostering innovation while underlining key differences in the policy approach of the Chinese leadership. Findings identify the areas of reform for India and also reveal that epistemic boundaries between India and China are beginning to blur so far as right-wing ideological regimentation is concerned, with possible implications for innovation.

## Introduction

As primary drivers of global growth, though rebounding from the pandemic to varying degrees, China and India as Asian giants are building themselves into knowledge economies for sustainable economic growth, socio-economic development, and success in the competitive global market. Both are beset with common development challenges such as poverty, income inequality, urban–rural inequality, and health hazards while operating in a global economic environment marked by the primacy of knowledge and the ascendancy of “innovation economics” (Terzic, 2018). Given the criticality of higher education institutions (HEIs) as “production sites of human capital” (Schulte, 2019) for driving knowledge economies, India and China are on the path to reforming their higher education systems. This development reflects the global phenomenon of the transformation of the role of postsecondary institutions from “cultivating gentlemen in Newman’s ideal, the Humboldtian promotion of science and scholarship, to Kerr’s teaching–research–service uses of the ‘multiversity’, and then to be a vital engine for the global knowledge economy” (Chen, 2012: 101).

Innovation, defined in terms of output, may be classified into “knowledge creation, knowledge impact, knowledge diffusion, intangible assets, creative goods and services, and online creativity” (Chiu et al., 2016: 370). Indeed, in the wake of technology-induced disruption of labor markets, “transformation of human capital” (Schwab and Zahidi, 2020) in terms of reskilling and upskilling and investment in innovation is indispensable. Arguably, innovation in totality results from human capital development, the level of economic development and economic size (Dutta et al., 2019), and a host of social, political/institutional, and cultural factors. Chiu et al. (2016) argue that in the face of weak institutional support, heavy investment in education or research and development (R&D) can facilitate, at most, *knowledge creation* and *knowledge impact* (the economic value of a country’s new products and services for that country itself) but not *knowledge diffusion* or transformational innovation (the influence of a country’s new products and services in the global innovation industry). As such, they place both China and India among Cluster 3 countries that are ahead of low-income countries in innovation performance but trail behind Cluster 4 countries (such as the United States, Canada, and Japan) in knowledge diffusion or global influence of their innovations.

Of late, India and China have improved their ranking in innovation. China maintains its 14th position in the Global Innovation Index (GII; World Intellectual Property Organization (WIPO), 2020) after making into the top 15 in 2019, and it is the only middle-income economy to figure in the top 30. India, “a lower middle-income economy,” at the 48th place made it to the top 50 for the first time in the GII 2020 edition. Notably, China and India have the world’s largest higher education systems in terms of student enrolments of 37 million and 34.5 million, respectively. Indian colleges and universities, numbering 51,649 in 2018–2019, have a gross enrolment ratio^1^ (GER) of 26.3, while China’s GER is 51.6. As for the reengineered focus of higher learning institutions, China’s 13th five-year plan (FYP) (2016–2020) gave a green light to “innovation-driven development” and entrusted its universities and research institutions with creating “national technological innovation centers.” And India’s National Education Policy (NEP; Ministry of Human Resource Development, 2020) underlines the imperative for higher education to be the foundation of “knowledge creation and innovation” to boost the national economy (pp. 33).

With the focus on India, this paper investigates how its political leadership has facilitated higher education reform for fostering innovation. Alongside, it underlines key differences in the policy approach of the Chinese leadership. Thus, the paper fills the deficit in comparative studies on HEIs in China and India (Bingman, 2010; Kapur and Perry, 2015; Perris, 2015; Reddy et al., 2016) that have neglected the knowledge economy aspect despite its significance in both countries’ economic and educational priorities. Besides, the rise of China and India has spawned literature on comparing the advancement of Chinese and Indian economies in innovation (Crescenzi and Rodríguez-Pose, 2017; Fan, 2011; Kennedy, 2016). But this segment misses the higher education component, which this paper addresses. Interestingly, China represents a monolithic political system whereas India is a parliamentary democratic republic, currently governed by the rightist party at the center. Typically, single-party governed states are known for totalitarian and ideological control over society, while liberal democratic regimes are associated with individual liberty and freedom of speech and expression. As such, qualitative inquiry is warranted into policy approaches and practices of both governments in promoting innovation led by universities.

### Limitations of the existing scholarship

Notably, the existing quantitative literature does not find necessary connections between democracy and innovation (Gao et al., 2017). Nor is there a statistically significant direct effect of democracy on economic growth (Baum and Lake, 2003). For example, Gao et al. (2017) employed a data set of 156 countries in the 1964–2010 period and found that democracy has “no direct positive effect on innovation measured with patent counts, patent citations and patent originality.” In their discussion of the political economy of growth, WeBaum and Lake (2003) take note of perspectives of “compatibility” and “conflict.”^2^ While the compatibility viewpoint sees democracy as an enabler of growth through limits to state intervention in economy, the conflict perspective posits that “redistributive policies” resulting from “populist pressures” in a democracy are inimical to growth.

At the micro-level, the democracy debate may be condensed into the ideology–innovation relationship especially in the context of private funding for higher education, fostering R&D, and the political commitment to allocation of resources, subsidies, and tax incentives (Dolfsma and Seo, 2013; Wang et al., 2019). In their study of 110 countries from 1995 to 2015, Wang et al. (2019) conclude that the right-wing ruling party tends to benefit capitalists who are “more willing to engage in R&D activities and more likely to promote the application of new technologies, thus stimulating the progress of technology” (pp. 1233). In this case, technical innovation reflects in trademark and patent applications. Conversely, leftist and central-leftist political parties have in-built reservations about new technologies for fear of possible adverse effects on the working class (Vivarelli, 2014; cited in Wang et al., 2019). Yet another perspective transcends rightist–leftist trappings or even the welfare state–capitalist state divide. Accordingly, the 21st century “innovation state” (Etzkowitz, 2018) strives to drive productivity through such measures as investments in science and technology and encouragement for the growth of new firms in collaboration with industry and university. And universities’ pivotal role in innovation occurs primarily through talent attraction and training, production of fundamental research for supporting “technological progress,” commercialization through patenting, and knowledge codification through publications (Watney, 2020). Be that as it may, it merits an investigation as to how Indian universities have fared in fostering innovation, especially when Indian policy makers tend to keep bigger economies, specifically China, in view in assessing India’s progress.

### Methodology and paper organization

It is a qualitative study that is comparative, descriptive, and analytical. The comparative aspect lies in underlining the key differences between India’s policy approach and that of the Chinese leadership. The descriptive-cum-analytical feature underpins the Indian leadership’s policies and practices related to innovation through HEIs. The data sources include semi-structured interviews with 10 university vice chancellors and 15 academics, government documents, reports, indices, news stories, and grey literature. The interviewees were from private and state universities in northern and northwestern India and were selected out of a pool of prospective participants based on their readiness to participate in the study. In addition to the content and thematic analysis, quantitative metrics were analyzed for comparison between both countries.

Accordingly, the first part of this paper provides a snapshot of quantitative metrics of innovation originating from Indian and Chinese universities, followed by a brief overview of the political economy of higher education reforms in both countries. The second part discusses the role of Indian government in facilitating innovations through HEIs through policy direction (Hoareau et al., 2013), regulatory framework, and funding. The regulatory framework’s support for universities’ “innovation capacity” presupposes the “level of autonomy” universities have, whether it be organizational (“university governance structures, legal frameworks for collaboration with private organizations and the use of IP”), academic, financial, or staffing (Reichert, 2019: 51). The third part focuses on implications of the Modi government’s policies and practices for innovation at Indian universities. The final part outlines major conclusions.

## Universities and innovation

As members of BRICS (Brazil, Russia, India, China, and South Africa), a grouping of emerging economies in the global economic architecture, India and China are commonly sized up in innovation race regardless of their current asymmetry. In fact, China’s Science Technology Exchange Center in its BRICS Innovation Competitiveness Report 2017 predicted a rise in India’s innovation competitiveness with the possibility to outpace China in 2025–2030 owing to the massive promotion of science and technology and entrepreneurship. Indian policymakers are striving to bridge the gap to actualize this potential. This endeavor extends to the domain of universities, in the context of which Table 1 presents key quantitative metrics of comparison between both countries.

**Table 1.** Key innovation metrics in comparing Indian and Chinese universities.

Metrics	Country		Source
	India	China	
World’s 100 Most Innovative Universities		4	Reuters
R&D researchers per million population	253 (2018)	1307 (2018)	World Bank
University–industry collaboration in R&D	45	29	Global Knowledge Index
Number of STEM graduates	2.6 million (2016)	4.7 million (2016)	World Economic Forum (2016)
Ranking in scientific publications	Third Number of publications: 48,998 (2018)	First Number of publications: 5,28,263 (2018)	*Economic Times*

R&D: research and development; STEM: science, technology, engineering, and mathematics.

**Figure 1.**
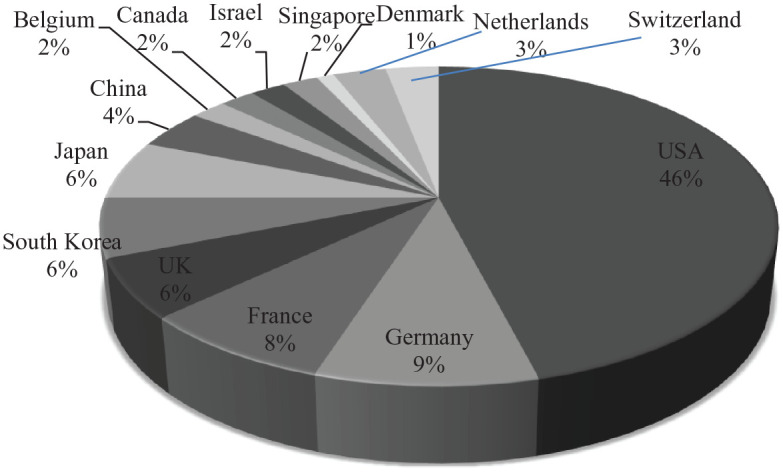
Share of countries in the world’s 100 Most Innovative Universities. *Source.* Prepared by authors using Reuters data (2019).

Reuters’s ranking of the World’s 100 Most Innovative Universities considers advancement of science, technology inventions, and powering of new markets and industries as markers of innovativeness. While it may not be the yardstick of innovativeness, it captures the dynamism of research universities contributing to novel technological solutions and original findings. The United States, Germany, France, and the United Kingdom are the top four hotspots, followed by South Korea, Japan, and China (Figure 1). China’s Tsinghua University, at the 41st place, filed 834 basic patents in the 2012–2017 period, with a 62.7% success rate in patent grants. And its commercial impact score (34.8) indicates “how often basic research originating at an institution has influenced commercial R&D activity, as measured by academic papers cited in patent filings” (Ewalt, 2019). Three other universities in China—Peking University, Zhejiang University, and Shanghai Jiao Tong University—figured in the list.

No Indian university clinched the spot because of the low level of patent filing by and commercial impact of Indian institutions. It is hardly surprising since in world university rankings as well, Indian universities are at the lower end of the ladder. Nevertheless, the laggardness of Indian universities is a reminder to Indian policymakers that China, India’s strategic competitor and a country with the nearly similar demographic size and socio-economic challenges, has swiftly and strenuously shaped its trajectory upward in the competitive arena of knowledge economy. This is especially noteworthy because just a little over the past decade, both countries shared the tag of “emerging technological powers” (Dahlman, 2007). Undeniably, Indian startup ecosystem has emerged as the world’s third largest, funded mainly by home-grown venture funds, old economy companies, and angel investors (Sengupta and Narayanan, 2019). But universities’ share in driving innovation is limited due to the lack of world-class research universities. A mere 8% of 892 Indian universities participated in patenting and received a total of 393 patents between 1958 and 2017 (Selvamani and Arul, 2019), although overall patent applications grew from 42,763 in 2014–2015 to 50,659 in 2018–2019, with the growth in patent grant from 14% to 30%.

Notably, on the Global Knowledge Index, China clinched the 29th spot and India 45th in university–industry collaboration in R&D. Furthermore, China is addressing the gap in Chinese universities’ share in R&D which is 7%, compared to the industry’s and the government’s contribution of 77% and 16%, respectively. For example, in 2019, R&D spending by Chinese HEIs increased by 23.2% compared to 2018 (Adams, 2021; Hu, 2020a). As such, China is radically shifting away from the categorization of universities in developing countries as typically being “training sites for knowledge workers rather than innovators” (Wu, 2018: 125). A related observation is that in India’s case, the central government and state governments’ combined share is dominant (51.8%) in gross expenditure on R&D (GERD), while the contribution of business enterprises and public sector industry is 37% and 4.6%, respectively, leaving a thinner slice for the higher education sector. In fact, 31% of the central government GERD is incurred by the Defence Research and Development Organisation (DRDO) alone. Thus, India’s democratic state has a disproportionate role in R&D compared to China’s communist state, although it may be argued that so far as R&D spending comes from state-owned enterprises, it constitutes an extension of the Chinese government’s role (ChinaPower, n.d.).

Technology transfer is considered yet another indicator of university-driven innovation. Although the rate of such transfer in India is not known, its level is known to be low, except for premium institutions such as Indian Institutes of Technology (IITs) at Delhi, Bombay, and Kharagpur. As for China, university technology transfer organizations in China were established as early as the 1980s to facilitate technology transfer (TT) between university and industry (Zhang et al., 2018). The imperative of high economic growth, recognized by China’s top leadership, underpinned such initiatives—as manifest from a string of measures such as Special Economic Zones (SEZs). Tsinghua University, for example, has eight types of TT organizations: original TT office, renewed TT office, university-run enterprises, university science parks, university-owned enterprises, national engineering research centers, university-region joint research institutes, and university-enterprise joint research centers (Zhang et al., 2018). And between 2000 and 2014, TT contracts with firms generated an average of nearly 13% of R&D revenues in higher education (Wu, 2018: 142). Nevertheless, it is estimated that technology transfer among China’s universities is less than 10% of the rate of foreign universities. The Chinese government is, therefore, keen on stepping up this rate, as described subsequently in this paper.

As far as the volume of scientific publications is concerned, India has built an impressive position with its global third rank, accounting for 48,998 publications in 2018. According to the *Economic Times*, China with its topmost position had 5,28,263 publications in the same year, partly because of vigorous international collaborations in authorship. Nevertheless, between 2008 and 2018, India had the fastest average annual growth rate of scientific publications with 10.73%, while that of China’s was 7.81% (*The Times of India*, 2018). Furthermore, India’s publication momentum has gathered speed to excel on a global level:

Indian researchers are publishing more than the global averages on key topics related to agricultural production, health and sustainable energy . . . 1.5 and 1.8 times the global average on smart-grid technologies, photovoltaics, biofuels and biomass and wind turbine technologies . . . The proportion of its research publishing output on climate-ready crops is triple the global average. Output is more than twice the global average on medicines and vaccines for tuberculosis, traditional knowledge, water harvesting, maintaining genetic diversity and pest-resistant crops. (Niazi, 2021)

As for citation impact, India had 235 frequently cited papers in 2011, which was 0.52% of its total science and technology paper output, while China had 1131 frequently cited papers, occupying the top slot among BRICS countries (Shan et al., 2017). Clearly, China outflanks India in innovation metrics. It is attributable to China’s fierce national ambition, its political culture of setting targets and pursuing them aggressively, and the continuity of the political regime allowing for quick reforms and uninterrupted implementation. In fact, higher education is pivotal to implementing China’s strategic plan to facilitate innovation-driven development. China’s national plan (2010–2020) underlines HEIs’ crucial role in the “state innovation system by encouraging them to contribute to innovation in knowledge, technology, national defense, and to regional innovation systems” (pp. 19–20). The crux of the policy intent is to transform China from an “innovation sponge” to an innovation leader.

### Political economy of higher education reforms

In the chronicles of history, from 1949 through the mid-1970s, higher education system in China largely catered to communist ideological indoctrination and to students’ technical training for serving the planned economy (Jain, 2019). Deng Xiaoping, leader of the People’s Republic of China from 1978 until 1989, set educational reforms in motion describing education as the “crucial basis for a drive towards economic and technological modernization” (Qiping and White, 1993: 410). This shift paved the way for the massification of higher education in alignment with the economic liberalization program to unleash the country’s productive forces.

China’s economic success story was noticeable between 2001 and 2010 when its gross domestic product (GDP) growth rate averaged 10.5%. Its impressive economic performance earned the epithet of the “Beijing Consensus,” though controversial, denoting vindication of the Chinese model of “authoritarian capitalism” in contrast to the Washington Consensus based on the “primacy of the market.” Over time, it became clear that the conventional growth model was unsustainable in the face of persisting poverty, “sagging exports,” regional inequality, environmental degradation, and growing social protests (Jain, 2017). Also, China’s “innovation imperative” stemmed from its aging population and the diminishing labor force as well the decline in the return on fixed asset investment (Woetzel et al., 2015: 9). Thus, the shift in China’s economic growth model through the 13th FYP underpinned the political economy of China’s higher education reforms. The 13th FYP outlined a new growth model which moves away from export-and-investment-reliant and carbon-based unsustainable growth and embraces technological innovation—further emphasized in the 14th FYP (2021–2025).

In India’s case, the political economy perspective posits that the post-independent, socialist democratic India’s “import substitution industrialization (ISI)” (Datta, 2017) policy demanded domestic human capital on a suitable scale. The state investment in basic and heavy industries required a consistent supply of engineers and technical personnel, which led to the creation of IITs and Indian Institutes of Management (IIMs) as autonomous technical institutes that received funding from the central government and emerged as centers of excellence (Datta, 2017). The policy focus on higher education rather than on primary education was especially evident between the early 1950s and the early 1970s (Kapur and Perry, 2015), generating more spending on higher education. As India integrated into the global economy, following the 1991 economic reforms and the global phenomenon of the “primacy of economy” in the neoliberal age, the imperative for injecting competitiveness into Indian economy was recognized. The National Knowledge Commission, a high-level advisory body to the prime minister, was formed in 2006 to pave the way for “knowledge-oriented paradigm of development.” One of its terms of reference was to build excellence in India’s educational system. Later, the National Innovation Council was set up by the United Progressive Alliance (UPA) government to help transform the country into an “innovation nation” and it contributed to creating “innovation ecosystems at universities through University Innovation Clusters” (National Innovation Council, 2013). Then Prime Minister Manmohan Singh further declared 2010–2020 as the decade of innovation, stating, “The time has come to create a second wave of institution building and of excellence in the field of education, research and capability building so that we are better prepared for the 21st century.”

Moreover, India’s 11th FYP (2007–2012) provided for establishing 14 world-class innovation universities in India (*DrEducation*, 2009). And the Science, Technology, and Innovation Policy (STIP, 2013) emphasized on “academia–research–industry partnerships” through “special and innovative mechanisms” and called for the “mobility of experts from academia to industry and vice versa.” Hence, a combination of measures and policies during the UPA regime aimed to place the country on the innovation-led growth track. The new regime, led by Prime Minister Modi, embarked on the innovation drive couched in the encompassing mantra of “atmanirbhar Bharat” or self-reliant India. Modi exhorted students to “innovate, patent, produce, and prosper” (*Economic Times*, 2020) and underlined innovation as the direction for a “New India.”

### Current regimes, universities, and innovation

Launched by the Modi government, India’s NEP, July 2020, takes cognizance of India’s research and innovation (R&I) investment that is mere 0.69% of GDP in comparison with 2.1% in China or 2.8% in the United States. It proposes for more investments in research to support India’s rise in “global knowledge production” (pp. 45). To address the missing focus of Indian universities on research and innovation, the policy provides for the National Research Foundation (NRF) with an “overarching goal” to infuse research culture in Indian universities and competitively fund research in multiple disciplines: science, technology, social sciences, and arts and humanities (pp. 45). The government announced in its 2021 budget allocation of Rupees 500 billion to the NRF for a period of 5 years.

In the words of K. Vijay Raghavan, Principal Scientific Adviser to the Indian government, the NRF will “seed, grow, and facilitate research at academic institutions, particularly at universities and colleges where research capacity is currently in a nascent stage.” Notably, teaching receives a disproportionate focus in most Indian universities, with less than 1% of the country’s nearly 40,000 HEIs engaged in research (Kumar, 2021) and R&D funding concentrated in government laboratories and premium institutes of excellence (Nature, 2021). This scenario is evident from India having a mere 253 researchers in R&D (per million people) in 2018, while China had 1307. In 2000, India had 110 such researchers while China had 539. Hence, both countries reported over a 50% increase, but China surpasses India by five times in size. Interestingly, even though India had 2.6 million STEM graduates in 2016, this volume does not necessarily translate into pursuit of or caliber for research careers. It is expected that the Prime Minister’s Research Fellows (PMRF) Scheme, announced in Budget 2018–2019, would encourage “meritorious” graduates to take up a career in research to contribute to “development through innovation.” Currently, these grants are being offered through 38 institutions in the country, including all the IITs and Indian Institutes of Science Education and Research (IISERs). India’s draft STIP also envisions doubling the number of full-time equivalent researchers every 5 years.

Furthermore, India’s National Intellectual Property Rights Policy (Government of India, 2016) lent impetus for universities to file more patents. It outlined such steps, relevant for postsecondary institutions, as the development of IPR expertise in academia, formulation of institutional IP policy/strategy, introduction of IP teaching, and strengthening IP Chairs in HEIs to provide “quality teaching and research” (pp. 18). In fact, IP protection has been recognized as the “first step” to boosting technology commercialization at Indian HEIs. As a result, patent filing has gained traction with universities setting up IPR cells and assisting students with legal counseling and filing paperwork (Viegas, 2019). Simultaneously, the Kalam Program for Intellectual Property Literacy and Awareness (KAPILA) is in place to disseminate information among higher education students and faculty about mechanisms and procedures involved in filing IP in India and globally.

Development of talent in artificial intelligence is yet another policy priority in both India and China. India’s NEP 2020 provides that universities offer doctorate and masters programs in machine learning and in multidisciplinary fields (“AI” + “X”). And China’s 2017 AI development plan establishes a distinct connection between AI talent and the country’s education system, outlining the following steps: building the “high-end talent team as the most important development of artificial intelligence”; and strengthening “the talent pool and echelon construction, especially to accelerate the introduction of the world’s top talent and young talent, and form China’s talent highland of artificial intelligence” (Jain, 2020). In April 2018, China launched the Artificial Intelligence Innovation Action Plan for Institutions of Higher Education, which stipulates that by 2030,

colleges and universities will become the main force behind building the world’s main AI innovation centers and will lead the development of a new generation AI talent pool to provide China with the scientific and technological support and guaranteed talent to put it at the forefront of innovation-oriented countries.

Hence, China’s proactive action reflects in moving from a generic AI plan to a detailed action plan focused on postsecondary education (Jain, 2020).

In this context, it is noteworthy that tests and memorization are virtually embedded in Indian and Chinese learning processes. Such entrenched practice detracts from innovation culture, leading policy makers to make assessment reforms. Thus, China has reoriented its national exam to incorporate “complex analytical skills” (Fuller, 2015) with a view to turning away from teacher-centric classrooms. Similarly, India’s NEP 2020 provides that assessment in India’s educational system will shift to a formative one and one that “tests higher-order skills, such as analysis, critical thinking, and conceptual clarity” (pp. 17). The necessity to build these skills by updating education curricula is tied to nurturing “innovation capability” in the age of knowledge economy. The Global Competitiveness Report 2020 reports that talent shortage owing to “outdated education systems” is acute across both developed and developing economies. In terms of graduates’ skill sets, it notes that Tanzania and China “are among the best improved, while India, Ethiopia and the United States have seen the largest decline” (Schwab and Zahidi, 2020: 22). Hence, the implementation of NEP 2020 should determine how well India aligns its education system with talent development.

Apart from policy launch, the Indian government has undertaken a plethora of innovation-specific measures. As a “flagship” initiative, the Atal Innovation Mission (AIM) has established “world-class” incubators at universities. Not losing sight of China’s phenomenal technological advancement, further manifest from its massive deep technology investment (such as in artificial intelligence and blockchain), India’s AIM Prime Program aims to promote “science based, knowledge intensive, deep technology entrepreneurship” through training and mentoring of researchers. And the National Innovation and Startup Policy for Students and Faculties, 2019, provides an extensive 10-point guiding framework for HEIs: setting up an “Innovation Fund” through allocation of at least 1% of the institution’s total budget; academic break to work on startups; and 2%–9.5% equity in startup by the institution’s incubator, apart from IP ownership mechanisms. Furthermore, the education ministry’s Innovation Cell is mandated to work with HEIs to promote startups and entrepreneurship. Its initiatives include Smart India Hackathon (SIH)—a platform for students to devise “world-class solutions” for the world’s top industrial organizations—and Institution’s Innovation Councils (IICs), established in HEIs in 28 states and 6 union territories, to create a “vibrant local innovation ecosystem.” IICs, set up without substantial capital investment, cover multiple fields such as pharmacy, management, medical sciences, art, science, and commerce, with a total of 483 incubation centers (Institution’s Innovation Council, n.d.).

As for the specific focus on building a US$100 billion bioeconomy by 2024, the government’s Innovate in India program fosters industry–academia collaborative research (BIRAC, n.d.). Notably, under the scheme of Bio-incubators Nurturing Entrepreneurship for Scaling Technologies, bio-incubation facilities have been set up at such institutions as the University of Hyderabad, Ahmedabad University, and Manipal Academy of Higher Education. Clearly, it is a top-down initiative like the aforementioned programs and schemes that dot the innovation landscape in India under the current leadership.

In fact, government intervention is integral to national innovation systems in both India and China in contrast to the US model of the predominance of industrial clusters in shaping the innovation system (Wonglimpiyarat and Khaemasunun, 2015). Li et al. (2020) sum up the Chinese government’s “dominant role” in terms of launching, funding, and controlling key innovation projects, owning nearly all major research institutions, setting up “primary innovative infrastructures” such as science parks and startup incubators, and formulating and reforming innovation and entrepreneurship policies and regulations (pp. 511). Similarly, the Indian government is a centrifugal force in fostering innovation competitiveness in higher education and industrial sectors, as manifest from a flurry of measures (Table 2). And like China’s vigorous pursuit of indigenous innovation, India’s science and technology policy, under formulation, lays such emphasis, although China’s embracement of indigenous innovation strategy dates to 2006 during President Hu Jintao’s leadership.

**Table 2.** Innovation measures for Indian higher education institutions, 2014–present.

Policies	Programs/Initiatives
National Education Policy	Atal Innovation mission
National Innovation and Startup Policy for Students and Faculties in Higher Education	National Research Foundation
National Intellectual Property Rights Policy	Smart India Hackathon
Science, Technology, and Innovation Policy (currently in the draft form)	Institution’s Innovation Councils
	The Global Initiative of Academic Networks in Higher Education
	Innovate in India
	KAPILA—Kalam Program for IP Literacy and Awareness

At the same time, the following key differences are found in policy approaches and determinants of Indian higher education reform in comparison with China’s under Xi Jinping’s leadership.

First, the Chinese government is steering the focus of Chinese universities away from patent volume to patent quality such as commercial viability. It has emphasized the strategic role of universities in the chain “from ideas to development and commercialization” through knowledge creation. With this, China is moving past the phase that India is currently in. Taking a cue from world-class universities, it is rather pushing for “invention evaluation.” According to the *China Daily*, the government guidelines in this context include the following:

- The government will assist in establishing IP management offices at “better-resourced” universities and in launching courses in IP management and technology transfer.
- Technology transfer, rather than the number of patent applications and patent grants, will carry more significance in performance appraisal.
- Universities have been asked to slash financial incentives for patent grants, with inventors to be rewarded with a bigger share of revenue resulting from commercialization (Hu, 2020b).

Governmental involvement in setting priorities for universities has intensified during the Xi regime to spur innovation. In positive terms, it is a corrective measure to curb “junk patents” that had spawned following the Ministry of Education (MOE’s) previous evaluation criteria for university researchers, namely, the number of patents, and the resulting pressure to meet performance targets (Cheng and Huang, 2016). In India’s context, university–industry technology transfer requires addressing a host of factors reported by administrators of Indian universities: lack of creativity and critical thinking in curricula; overemphasis on publications due to lack of awareness on patenting and commercialization; inactive IP cells at universities; lack of qualified people to manage IP/ technology transfer activities; and conflict between commercially viable and academic research (Ravi and Janodia, 2022). Hence, simply creating IP cells will not be sufficient. Overall, commercialization is a relatively new notion for most Indian universities which have been embedded into the teaching layer of higher education.

To provide fillip to university–industry collaboration, Innovation and Science @Bharat, division of the Office of Principal Scientific Adviser (PSA) to the Government of India, is working toward forming partnerships between academia and industry in the form of joint R&D, establishment of Centers of Excellence (CoEs) by industry in academia, and innovative solutions for social benefit. Interestingly, analysis of the PSA-released list of outcomes shows (1) the Indian government’s predominant presence in these collaborations (other than that of foreign companies) with a minuscule share of domestic private companies, and (2) the major role of IITs, which indicates a need for diversification supported by research capacity across non-IITs. For example, National Buildings Construction Corporation (NBCC) Limited, a government company, will set up a lab space as a CoE at IIT Madras campus, and IITs’ R&D proposals have been shortlisted by Bharat Electronics Limited, an Indian state-owned company. Interestingly, the PSA list includes the Election Commission of India’s acceptance of an IIT proposal as an example of industry–academia partnership (Innovation and Science @Bharat, n.d.).

Second, while India has attempted to catch up with China, the latter has been driven by tech-nationalism to replace the United States as the foremost superpower in science and technology. President Xi (2017) strives to “cultivate a large number of world-class scientists and technologists in strategically important fields, scientific and technological leaders, and young scientists and engineers, as well as high-performing innovation teams” (pp. 27). He has set out to create “a country of innovators,” emphasizing the centrality of science and technology as a path towards modernity” (Xi, 2017: 27). To Xi, the sphere of higher education is an integral part of the battle of advanced ideas and skills in an increasingly competitive marketplace with little constraints from rival nation-states. Hence, while India sets out to address deficiencies, whether through IP filing, or incubation centers, in the spirit of “made in India,” China leaps up to newer avenues. And in view of the technological war with the United States, development of indigenous capabilities has assumed more significance for Chinese HEIs to spearhead the shift from “made in China” to “created in China” (*Global Times*, 2021). This reflects from two major decisions of the Chinese education ministry in 2020–2021: establishing centers on quantum science, brain science, synthetic biology, aerospace engineering, and next-generation mobile communication technology at 14 universities, and issuing a list of 12 new technology schools for cultivation of “cutting-edge” research talent.

Third, while China has an official blueprint dedicated to AI for postsecondary institutions, India does not have an exclusive action plan to revamp HEIs for AI readiness. Also, the Indian policy document (2018) provides a broad-brush direction for course upgrades and training in AI, whereas China’s 2018 action plan carries AI training-specific targets for 2020, including the development of 100 “AI + X” majors for interdisciplinary growth, the publication of 50 world-class undergraduate and graduate textbooks, the establishment of 50 AI schools, and the launch of 50 national-level 20 quality open online AI courses (Jain, 2020). Impliedly, China is more aggressively pursuing talent development in AI through higher education reforms.

Fourth, China has been more proactive in reversing brain drain and in attracting foreign talent to support and trigger innovation. Following the strong government endorsement, in October 2017, Shanghai-based universities such as Fudan University, Shanghai Jiao Tong University, and Shanghai University dispatched a 12-university delegation to American universities such as Harvard University, the University of Pennsylvania, and Columbia University in hunt for skilled graduates and professors (Sharma, 2018). Premier Li Keqiang stated at the National People’s Congress (NPC) session in March 2018, “To spur innovation, China will make more effort in encouraging overseas Chinese students to return after completing their studies, while creating a fast track program to attract more foreign talent to China” (Sharma, 2018). Notably, China has launched numerous programs toward this end from time to time such as the Hundred Talents Program (1994), 111 Program (2005), Thousand Talents Program (2008), and Ten Thousand Talents Program (2012). However, one of the limitations is that irrespective of the massive funding allocation for these programs, returnees have faced such issues as a heavy reliance on personal relations to secure funding (Chiu et al., 2016).

**Figure 2.**
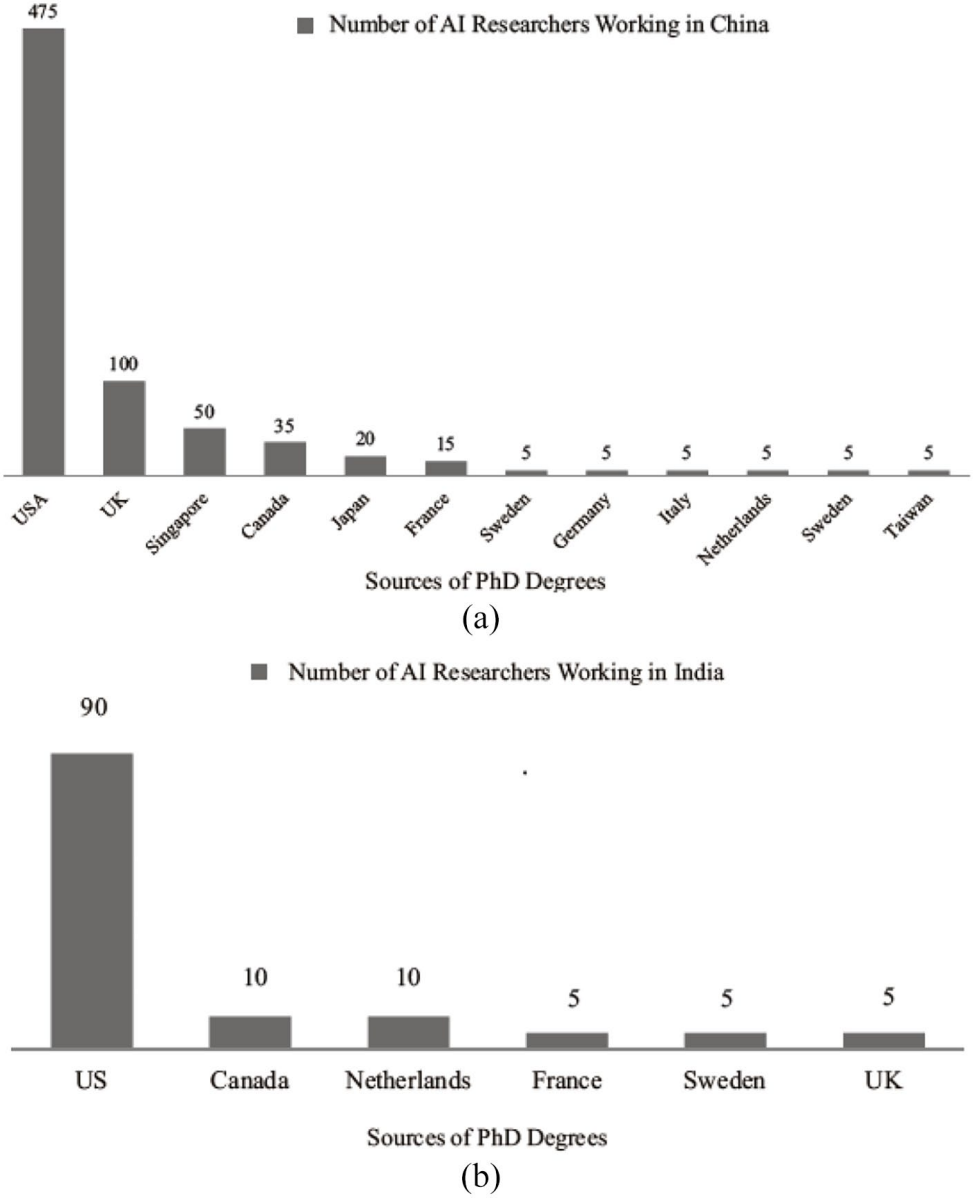
(a) AI talent inflow to China. (b) AI talent inflow to India. *Source*: Consulted the 2019 Global Talent Report data.

Importantly, China launched the “Double First-Class University Strategy” in 2015 as it strives to advance an “innovation excellence culture” in science. This ambitious initiative sets out to optimize the disciplinary structures of major research universities, and intensify their efforts to bring in top scientists and talented researchers from within China and abroad. The authorities identified 42 out of 2000 Chinese universities as part of “world-class” universities, and another 95 institutions to develop “world-class” disciplines. Because most of the designated schools are in well-developed metropolises and provinces, this policy appears to have perpetuated structural imbalances in the state’s allocation and distribution of public resources (Peters and Besley, 2018). Impliedly, China’s overseas talent attraction policy is not without gaps. But India is yet to establish itself as an attractive destination for competent researchers and scholars from abroad. Figure 2(a) and (b) presents an example of the gap in foreign talent flow into China and India.

As for India, the government’s Institutions of Eminence promotion scheme provides for hiring foreign faculty to address the deficit in talent attraction. Furthermore, the Global Initiative of Academic Networks in Higher Education, 2015, seeks to harness

the talent pool of scientists and entrepreneurs, internationally to encourage their engagement with the institutes of Higher Education in India so as to augment the country’s existing academic resources, accelerate the pace of quality reform, and elevate India’s scientific and technological capacity to global excellence. (Global Initiative of Academic Networks in Higher Education, 2015)

But India’s prime challenge is to attract top faculty especially given the disincentive of low academic salaries. Drawing a comparison with China on this front, Altbach and Mathews (2019) explain,

Indian salaries in the IITs, according to the latest Pay Commission recommendations, start at US$17,622 for assistant professors, rising to around US$38,165 for full professors. Higher ranks earn somewhat more. China, which is also actively luring top international faculty to its research universities, offers salaries of US$100,000 or more, along with additional research funding. (“The challenges” section)

Of late, institutions like IITs have been offering a higher salary package plus benefits to recruit faculty of Indian origin, but it may not still be enticing for those who have better opportunities overseas.

## Politics and higher education: implications for innovation in India

The previous sections illuminated differences in policies and operation of Indian and Chinese political regimes in terms of impacting HEI-induced innovations. With this, the areas of improvement for India were brought to light. However, while policy directives, funding, and schemes may be considered tangible measures to promote innovation, the intangible aspect of state support manifests in the government’s grant of autonomy and its policy practices. And this is why, given the paper’s focus on India, this section highlights practical challenges for India. Based on news stories and semi-structured interviews with university vice chancellors and academics, it presents themes on politics-based implications for innovation in India.

### Ambiguity and irrationality

There is a mismatch between policy and practice which is unfavorable to fostering innovation culture in Indian HEIs. NEP 2020 provides for a shift in student assessment to test “higher-order skills, such as analysis, critical thinking, and conceptual clarity” (p.17). But Indian policymakers’ statements detract from the purported national drive for a “scientific spirit.” For example, Indian Environmental Minister Prakash Javedkar’s refutation of a link between lifespan and air pollution drew flak from the World Health Organization (*Times Now*, 2019). As some academics interviewed for this paper put it in their analysis of the new education policy,

*Authors of the new education policy borrowed from the mainstream terminology to direct higher education reforms, and cabinet ministers’ language smacks of non-rational advocacy of the rightist agenda.* (Assistant Professor of Education, private university)

*The policy document refers to ancient Indian practices without providing solid connections between “what to achieve” and “how to achieve.” For example, it speaks of restoration of India’s role as Vishwa Guru and in this context mentions building India as a “global study destination.” This is an egregious simplification of what “Vishwa Guru” [world leader in learning] means.* (Senior Research Fellow, Social Sciences Department, private university)

*Innovation demands not just funding or infrastructure but also mindset; even if the government channels funds into research projects, it is at the same time promoting one line of thinking which if it becomes entrenched will stifle unconventional approach to problem solving. The implications may not be felt immediately but the seed of passivity and obedience to authority is being sown.* (Professor of Political Science, state university)

Some interviewees found fault with the implicit rhetoric in the Indian government’s policy postures. It was argued that schemes and measures to boost innovation are a glitter that conceal the tightening state control over information dissemination:

*It sounds impressive to see [projects such as] Digital India, Make in India, Startup India, and ATAL Innovation Mission. And maybe many more are on the anvil. At the same time, the government is tightening sinews on freedom of expression. For instance, digital media curbs, social media surveillance, as they do in China. So, basically it’s telling us to prepare the innovative dish by using the state-provided recipe, ingredients, and utensils.* (Professor of Political Science, state university)

*Actually, the government is serving old wine in a new bottle. It did away with the National Innovation Council that envisioned creation of the India Inclusive Innovation Fund to generate social impact of innovative projects. The current IMPRINT is a rehash of those ideas. To citizens it won’t matter of course insofar as the promised outcomes are realized.* (Professor of Economics 2, private university)

In light of above comments, it may be added that the Indian government’s attempt to shape scientific research along the ancient knowledge is evident. Its push for “panchgavya”^3^ to foster socially relevant innovation has triggered “backlash” among a section of scientists who advocate neutrality and objectivity in conducting research that allows for refutation of hypotheses sanctifying traditional knowledge. Jayant Murthy, a professor at the Indian Institute of Astrophysics, comments, “While the Chinese are on the far side of the moon, Indians are busy treating cancer with cow urine and looking to the past for modern fighter jets” (Murthy, 2019).

### Sinicization of the Indian higher education system

Higher education acts as the “vanguard” of the Chinese communist regime by functioning as an ideological enterprise that enables state supervision and control, facilitates indoctrination, ensures the “acquiescence” of intellectuals, and supports research in indigenous “discourse power” (Perry 2015). Ideational regimentation involves circumscribing the frontiers of discourse, information dissemination, research, and knowledge using regime-sanctioned ideological and cognitive concepts such as the sinicization of Marxism. Control mechanisms have especially tightened in the reign of President Xi Jinping who has instructed China’s higher learning institutions to “shoulder the important tasks of studying, researching and publicizing Marxism, as well as training builders and successors of the socialist cause with Chinese characteristics” (Ramzy, 2014).

This study suggests a gradual sinicization of India’s higher education practices with the ruling government’s fostering of a uni-linear thinking. It appears as if the Indian government is not just following China’s roadmap to scientific innovation but it is also emulating its academic control mechanisms. For example, in 2020, Peking University announced that academics and researchers attending overseas online conferences needed approval from university authorities in advance. Afterwards, the Indian government put in place the requirement for India’s public universities to seek clearance from the Ministry of External Affairs for holding online international conferences or seminars that deal with politically sensitive issues, and it added that participants’ names will require advance approval from the government.

From another perspective, V.N Rajashekar, member of the Save Education Committee, points out that educationists and universities had not been consulted in the design of NEP 2020, rendering the process undemocratic:

In a vast country like India with a lot of socio-economic, cultural disparities and varieties of colleges, universities and courses, evolving a national education policy is a gigantic task. Taking into consideration all these diversities, a professional committee of educationists, teachers and social scientists has to conduct an objective study and evolve scientific solutions; this democratic task, the drafting committee of NEP has not undertaken. So, the draft NEP is a mere compilation of the ideas of pro-government thinkers. (Thakur, 2021)

Infringement on institutional autonomy is another corollary. Nuclear scientist Anil Kakodkar’s resignation as the Chairman of the Board of Governors of the Indian Institute of Technology, Bombay, because of government interference is a case in point. Undeniably, the issue of the lack of autonomy is not novel. There has had been a demand for university autonomy in filling academic posts and in determining the fee structure (Mishra, 2010). But in the current dispensation, governmental interference is more than a web of rules and regulations, causing deterioration of the standards in faculty and staff selection with pronouncedly arbitrary recruitment decisions. Evidence suggests the escalation in recruitment on the basis of party affiliation at the cost of merit, with implications for quality in teaching and research. This compounds the existing faculty shortage situation. According to the All India Survey on Higher Education, the student enrolment in higher education institutes grew from 32.3 million in 2013–2014 to 36.6 million in 2017–2018, but the total number of teachers declined from 13,67,535 to 12,84,755 (cited in *India Today*, 2019). The 24:1 ratio in India is lower than 19:1 in Brazil and China. The Ministry of Human Resource Development (MHRD) report comments,

A low student-teacher ratio indicates the burden on a single teacher of teaching multiple students as well as the lack of time that each student gets. Apart from this simplistic effect, in an institution of higher learning, a smaller number of overburdened teachers are also unable to pursue any research or encourage their students to do so. (Cited in *India Today*, 2019)

It further points out that as a result, “the culture of questioning and reasoning cannot be inculcated as a part of higher education in most institutions.” Even though the HRD minister called for filling vacancies on a “war footing” level, the solution appears even more intricate than the problem owing to politicization of selection procedures (Lau, 2020). Gill and Singh (2020) comment,

the implicit eligibility criterion for vice-chancellorship at Indian universities as the ruling party’s or its parent body’s affiliation has marked a rush of entry of the right-wingers into the intellectual spaces in the country. One such example has been Gajendra Chauhan’s appointment as Film and Television Institute of India (FTII) chairman, triggering a 139-day protest by students, questioning Chauhan’s qualifications.

Furthermore, a common theme emerging from interviews with vice chancellors (VCs) and scholars is the diminishing academic freedom and autonomy:

*India has had a culture of debate since ancient times. And the people of Indian origin are heading MNCs as CEOs which testifies to their strategic entrepreneurial acumen. But in terms of quantity, we are not generating enough mass of analytical human capital base, thanks to the faulty teaching methods and outdated curricula. There is not much debate, discourse, and practical activities that could train young minds along problem-solving lines. Apparently this is beginning to change because there is a realization among policy makers and education providers that 21*^st^ *century skills are indispensable. The question is: where to get such faculty for non-IIT institutions . . . and another question is: how to cultivate critical thinking when dissent is curbed in the current right-wing regime. When you [the central government] can’t tolerate student activism, how can you tell them to think critically?* (VC 1, private university)

*There is a steady decline of whatever autonomy we had because of increasing political interference. The new policy is a sham.* (VC 2, private university)

*The so-called emphasis on critical thinking is that anti-minority thinking is critical thinking and any thought of inquiry challenging the current regime is considered anti-nationalist.* (VC 3, state university)

*If, as you [X] mentioned, India is taking the China road of ideological control, it’s possible that innovations would at most be incremental, not radical. It’s very common these days to speak of China’s success in the e-commerce and digital world as evidence of their capacity for innovation but they aren’t known for technological breakthroughs with global effect.* (VC 4, private university)

By giving China’s example, VC 4 noted the implications of ideological control for the prospect of breakthrough innovations in India. However, a different perspective was offered by another stream of academics:

*Has China been encumbered by ideology? No. It’s innovating on a consistently improved scale, whether it is patents, business models, or high-speed rail. Whatever controls exist they mostly impact humanities, and innovation, which China is aiming at, derives from STEM. Similar is the case with India: cultural resurgence might be the government’s agenda but that won’t impede innovation.* (Professor of Management, state university)

*The government is withdrawing from most sectors of governance . . . The whole atmosphere is tipped in favor of privatization, and this is liberating for a knowledge economy because with the government interference gone, individual initiative for innovation will receive encouragement.* (VC 5, state university)

*This government is taking the job of catching up with and even surpassing China very seriously. The Made in India campaign extends to manufacturing and virtually everything. So, we see the focus of innovation on indigenous knowledge, and this is how the government has provided much-needed thrust to relevant innovations that match with our local conditions. The [ National Intellectual Property] NIP policy is in place; artificial intelligence in education has received lots of attention; and the prime minister’s speeches are laden with the word “innovation” in his address to the youth. Results will take time to show but the beginning has been made.* (Professor of Economics 1, private university)

These excerpts suggest that some academics are positive about the government’s innovation practices and perceive a direct connection between the government’s privatization drive and a liberating environment for innovation.

## Conclusion

The foregoing discussion revealed that China is ahead of India in quantitative metrics of university-driven innovation because of massive reforms in higher education. In fact, China has unseated the United States, the world’s “leading powerhouse,” from its foremost position in patent applications. As such, the gap between India and China in innovation should not spring any surprises. However, India is catching up with several significant policies, action plans, and initiatives, with the National Research Foundation being among prime examples. Basically, while China’s proactive pursuit of innovation is encompassed in its Chinese Dream of national rejuvenation, India’s initiatives have been presented in the government’s stated goal of building a “self-reliant India.” What is, however, significant is that epistemic boundaries between India and China are beginning to blur so far as right-wing ideological regimentation is concerned.

This paper addressed the research question as to how the divergent political regimes have facilitated their countries’ innovation drive. Parallels between current regimes in India and China may be drawn as the former is exhibiting ideological controls, threatening the culture of dissent in the democratic India. While China’s technology giant Tencent is considered a world-class innovative company, the government’s crackdown on Alibaba is perceived as being prompted by its CEO Jack Ma’s criticism of financial regulators. Not surprisingly, the fact that he had to step down as president of the Hupan Innovation Center reinforces the upper hand of the communist state. As such, critics apprehend the repercussions of authoritarianism in the democratic India if it goes unchecked.

Arguably, Chinese universities’ achievements in innovation are largely driven by “top-down” initiatives and the financial succor of the communist regime. And innovation has had thrived in other non-democratic regimes as well such as the former Soviet Union. The crux of this analysis is not as to which political system is favorable to reforming higher education systems for innovation. What is significant to note is that while the liberal democratic environment has not yielded any distinctive advantage in favor of innovation in India, as evident from the leading position of Chinese universities, the growing political controls in India are sounding the death knell of creative potential. The political regime in India appears to be sanctioning the innovation-frame-of-mind selectively, which is applicable to encouraging socially relevant products and solutions rather than to the education system that produces a generation of critical thinkers. This trend has implications for nurturing innovativeness and scientific temper regardless of the new education policy or IP regime being in place. Hence, the acid test of the newly created National Research Foundation will lie in funding projects of promise without politicization of the research mandate.

It is pertinent to mention the trend for research-intensive universities to enter into R&D partnerships with international enterprises with the onset of globalization of R&D (Ma, 2019). For instance, Singapore is involved in developing western China through such projects as National Technological University of Singapore’s Entrepreneurship and Innovation Base in Yubei and the Sino-Singapore Guangzhou Knowledge City (SSGKC) project; another example is the Guangdong Technion–Israel Institute of Technology (GTIIT) (Eizenberg, 2020). Future research could investigate the role of foreign partners in fostering innovation ecosystems—inclusive of local industry and universities—and talent development in both countries.

## Notes

1. Total enrolment in higher education as a percentage of the population in the age group of 18–23 years.

2. As labeled so by Sirowy and Inkeles (1990).

3. Products prepared by a combination of constituents derived from a cow: milk, curds, ghee, urine, and feces.
